# Supplementary material for: Developmentally programmed nuclear pore complex replacement enables oocyte specification
Source: bioRxiv. 2026 Feb 16:2026.02.13.705775. Preprint. [Version 1] doi: 10.64898/2026.02.13.705775 (PMC12934936; doi:10.64898/2026.02.13.705775)
Supplement: Supplement 2 [file NIHPP2026.02.13.705775v1-supplement-2.pdf]

bioRxiv preprint doi: https://doi.org/10.64898/2026.02.13.705775; this version posted February 16, 2026. The copyright holder for this preprint (which was not certified by peer review) is the author/funder, who has granted bioRxiv a license to display the preprint in perpetuity. It is made available under aCC-BY 4.0 International license.

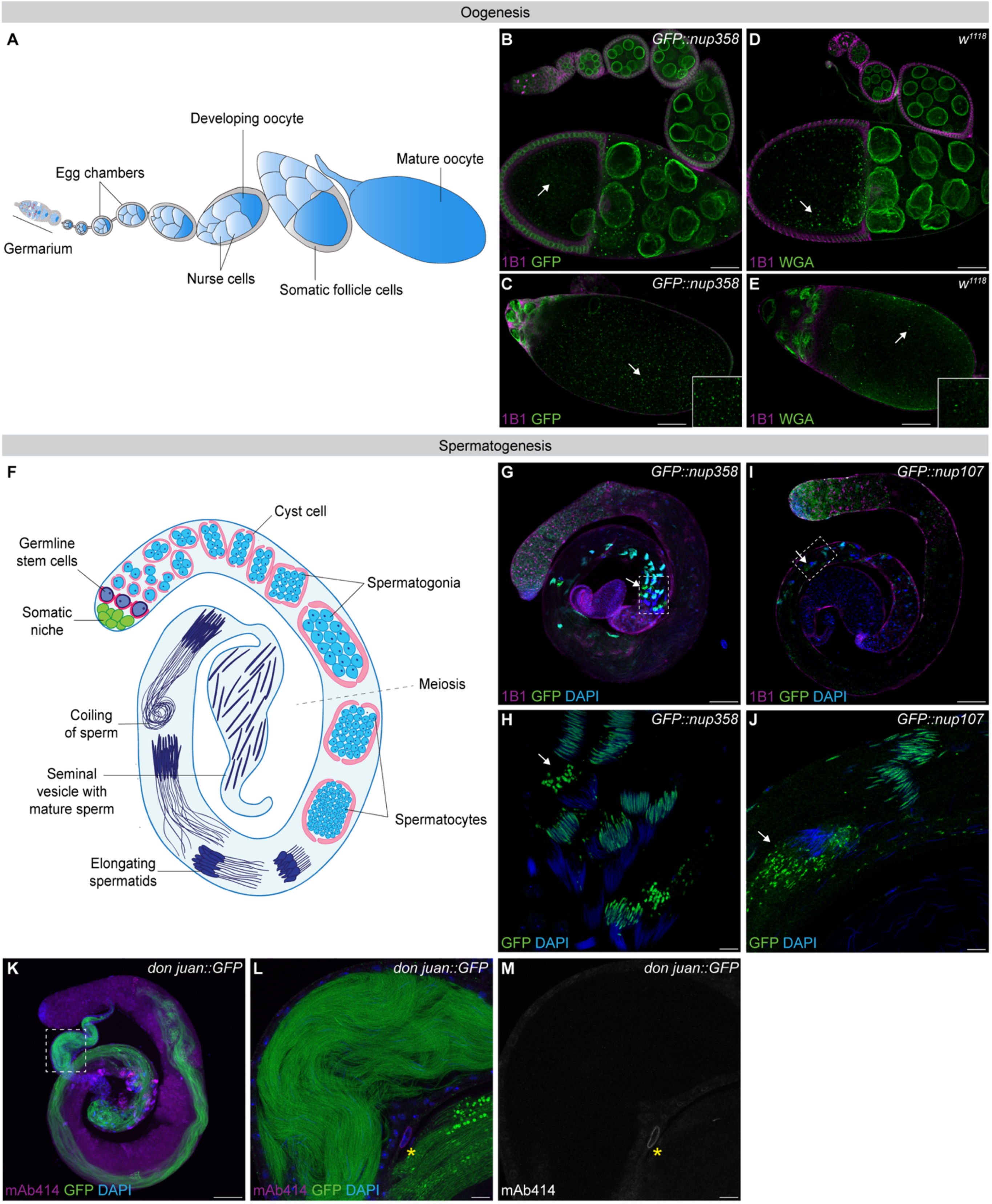

Venkat_fig.1S1

Figure 1S1. NPC and nucleoporin marker signal accumulates in oocytes but is largely absent from mature sperm.

bioRxiv preprint doi: https://doi.org/10.64898/2026.02.13.705775; this version posted February 16, 2026. The copyright holder for this preprint (which was not certified by peer review) is the author/funder, who has granted bioRxiv a license to display the preprint in perpetuity. It is made available under aCC-BY 4.0 International license.

(A) Schematic of the *Drosophila* ovariole.

(B) Confocal image of a *GFP::nup358* ovariole stained for 1B1 and GFP. Arrow indicates accumulation of GFP::Nup358 signal within the ooplasm of the developing oocyte.

(C) Confocal image of a stage 12 egg chamber showing accumulation of GFP::Nup358 in the ooplasm (arrow).

(D) Confocal image of a w^{1118} ovariole stained for 1B1 and wheat germ agglutinin (WGA); arrow indicates accumulation of NPC marker signal in the ooplasm of the developing oocyte.

(E) Confocal image of a stage 11 egg chamber showing ooplasmic accumulation of NPC marker signal (arrow).

(F) Schematic of the *Drosophila* testis.

(G) Confocal images of testis expressing *GFP::nup358*. White dotted boxes indicate late-stage spermatids in which the nucleoporin signal is not associated with DNA.

(H) Enlarged inset from (G) showing loss of GFP::Nup358 signal from late spermatids (arrow). Scale bar, 10 µm.

(I) Confocal images of testis expressing *GFP::nup107*. White dotted boxes indicate late-stage spermatids in which the nucleoporin signal is not associated with DNA.

(J) Enlarged inset from (I) showing loss of GFP::Nup107 signal from late spermatids (arrow). Scale bar, 10 µm.

(K) Confocal image of *don juan::GFP* testis stained for GFP and mAb414. White dotted box indicates the seminal vesicle containing mature sperm.

(L) Enlarged inset from (K) showing mature sperm in the seminal vesicle lacking detectable NPC marker signal. Scale bar, 10 µm.

(M) Grayscale channel illustrating absence of mAb414 signal in mature sperm; yellow asterisk marks a neighboring somatic cell serving as an internal positive control for NPC marker staining.

Scale bars, 100 µm unless otherwise indicated.

bioRxiv preprint doi: https://doi.org/10.64898/2026.02.13.705775; this version posted February 16, 2026. The copyright holder for this preprint (which was not certified by peer review) is the author/funder, who has granted bioRxiv a license to display the preprint in perpetuity. It is made available under aCC-BY 4.0 International license.

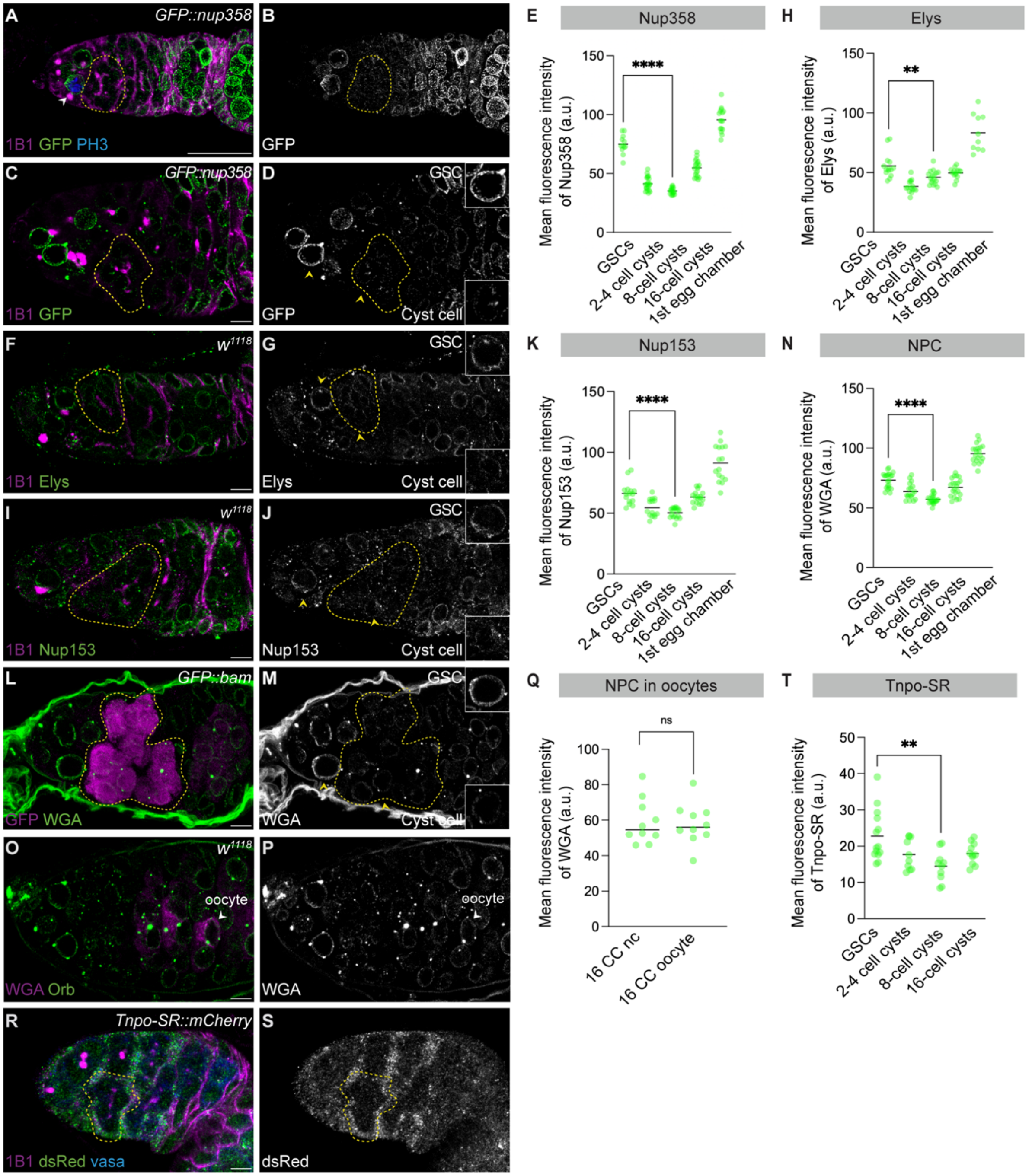

Venkat_fig.1S2

Figure 1S2. NPC marker signal is reduced during cyst differentiation independent of cell division and nuclear envelope breakdown.

(A–B) Confocal images of *GFP::nup358* germaria. Scale bar, 25 µm.

(C–D) Confocal images of *GFP::nup358* germaria highlighting NPC marker signal in GSCs and cyst cells.

bioRxiv preprint doi: https://doi.org/10.64898/2026.02.13.705775; this version posted February 16, 2026. The copyright holder for this preprint (which was not certified by peer review) is the author/funder, who has granted bioRxiv a license to display the preprint in perpetuity. It is made available under aCC-BY 4.0 International license.

Scale bar, 5 µm.

(E) Quantification of mean GFP::Nup358 fluorescence intensity (a.u.).

(F–G) Confocal images of w^{1118} germaria stained for Elys. Scale bar, 5 µm.

(H) Quantification of mean Elys fluorescence intensity (a.u.); n = 5 germaria; n = 5 germaria; ** = p = 0.0057.

(I–J) Confocal images of w^{1118} germaria stained for Nup153. Scale bar, 5 µm.

(K) Quantification of mean Nup153 fluorescence intensity (a.u.).

(L–M) Confocal images of *bam::GFP* germaria showing NPC marker signal in differentiating cysts. Scale bar, 5 µm.

(N) Quantification of mean WGA fluorescence intensity (a.u.).

(O–P) Confocal images of w^{1118} germaria stained for the oocyte marker Orb; arrowheads indicate oocytes. Scale bar, 5 µm.

(Q) Quantification of mean WGA fluorescence intensity in oocytes (a.u.); n = 5 germaria; ns = p = 0.8258.

(R–S) Confocal images of *Tnpo-SR::mCherry* germaria. Scale bar, 5 µm.

(T) Quantification of mean Tnpo-SR fluorescence intensity (a.u.); n = 5 germaria; ** = p = 0.0015.

Yellow dotted lines indicate 8-cell cysts. Statistical analysis where applicable was performed using a two-tailed unpaired Welch's t-test on mean fluorescence intensity. n = 5 germaria; **** = $p < 0.0001$ unless otherwise indicated.

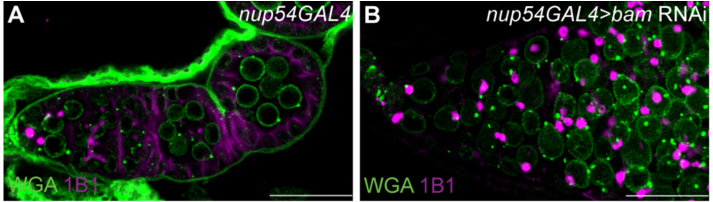

Figure 3S1: Nup transcription is active in the GSCs.
(A-B) Confocal images of *nup54GAL4* (control) germaria and *nup54* GAL4 driving *bam* RNAi. RNAi germaria phenocopy germline *bam* depletion, showing accumulation on GSCs/cystoblasts. This indicates that the *nup54* promoter is active in the GSCs/cystoblasts. Scale bars: 25 µm.

bioRxiv preprint doi: https://doi.org/10.64898/2026.02.13.705775; this version posted February 16, 2026. The copyright holder for this preprint (which was not certified by peer review) is the author/funder, who has granted bioRxiv a license to display the preprint in perpetuity. It is made available under aCC-BY 4.0 International license.

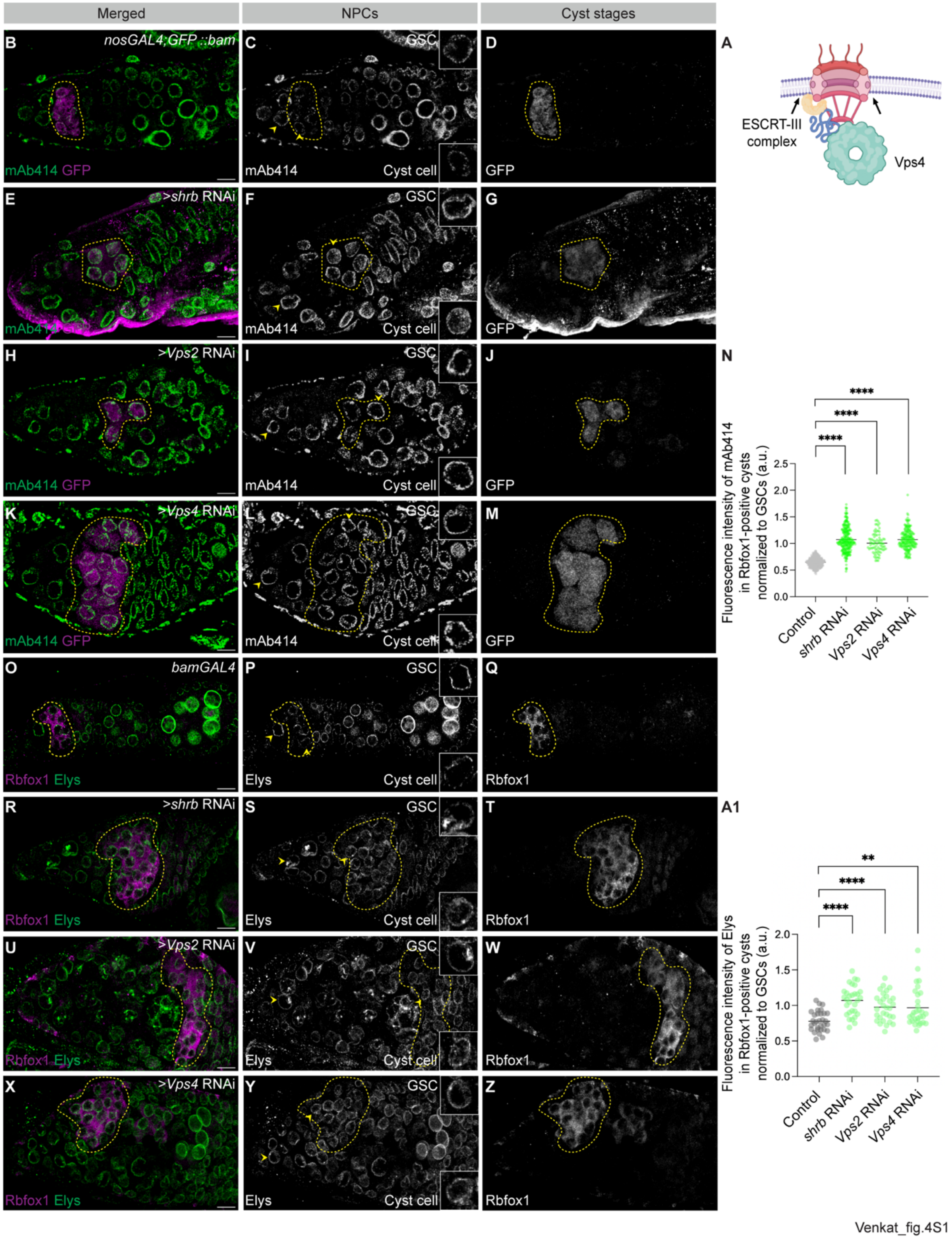

Figure 4S1: ESCRT-III/Vps4 machinery mediates reduction of nucleoporin levels during cyst differentiation.

(A) Schematic representation of the proposed ESCRT-III/Vps4-dependent pathway for NPC degradation. **(B–M)** Confocal images of control (*nosGAL4;GFP::bam*) and germline-specific depletion of *shrb*, *Vps2*, or *Vps4* in germaria, stained with mAb414 to label FG-repeat nucleoporins. GFP marks differentiating cysts. GSCs are indicated by yellow arrowheads, and yellow outlines denote 8-cell cysts. Scale bars, 10 µm.

bioRxiv preprint doi: https://doi.org/10.64898/2026.02.13.705775; this version posted February 16, 2026. The copyright holder for this preprint (which was not certified by peer review) is the author/funder, who has granted bioRxiv a license to display the preprint in perpetuity. It is made available under aCC-BY 4.0 International license.

(N) Quantification of mAb414 fluorescence intensity in GFP-positive cysts, normalized to GSCs within the same germarium and expressed in arbitrary units (a.u.). Depletion of *shrb*, *Vps2*, or *Vps4* results in a significant increase in nucleoporin signal relative to control. Statistical analysis was performed using a two-tailed unpaired Welch's *t*-test. $n > 5$ germaria per genotype; ****, $p < 0.0001$.
(O–Z) Confocal images of control and *shrb*-, *Vps2*-, or *Vps4*-depleted germaria stained for Elys. GSCs are indicated by yellow arrowheads, and yellow outlines mark Rbfox1-positive 8-cell cysts. Scale bars, 10 μm.
(A1) Quantification of Elys fluorescence intensity in Rbfox1-positive cysts, normalized to GSCs within the same germarium and expressed in arbitrary units (a.u.). Depletion of *shrb*, *Vps2*, or *Vps4* leads to a significant increase in Elys signal compared to control. Statistical analysis was performed using a two-tailed unpaired Welch's *t*-test. $n = 5$ germaria per genotype; ****, $p < 0.0001$; **, $p = 0.0012$.

bioRxiv preprint doi: https://doi.org/10.64898/2026.02.13.705775; this version posted February 16, 2026. The copyright holder for this preprint (which was not certified by peer review) is the author/funder, who has granted bioRxiv a license to display the preprint in perpetuity. It is made available under aCC-BY 4.0 International license.

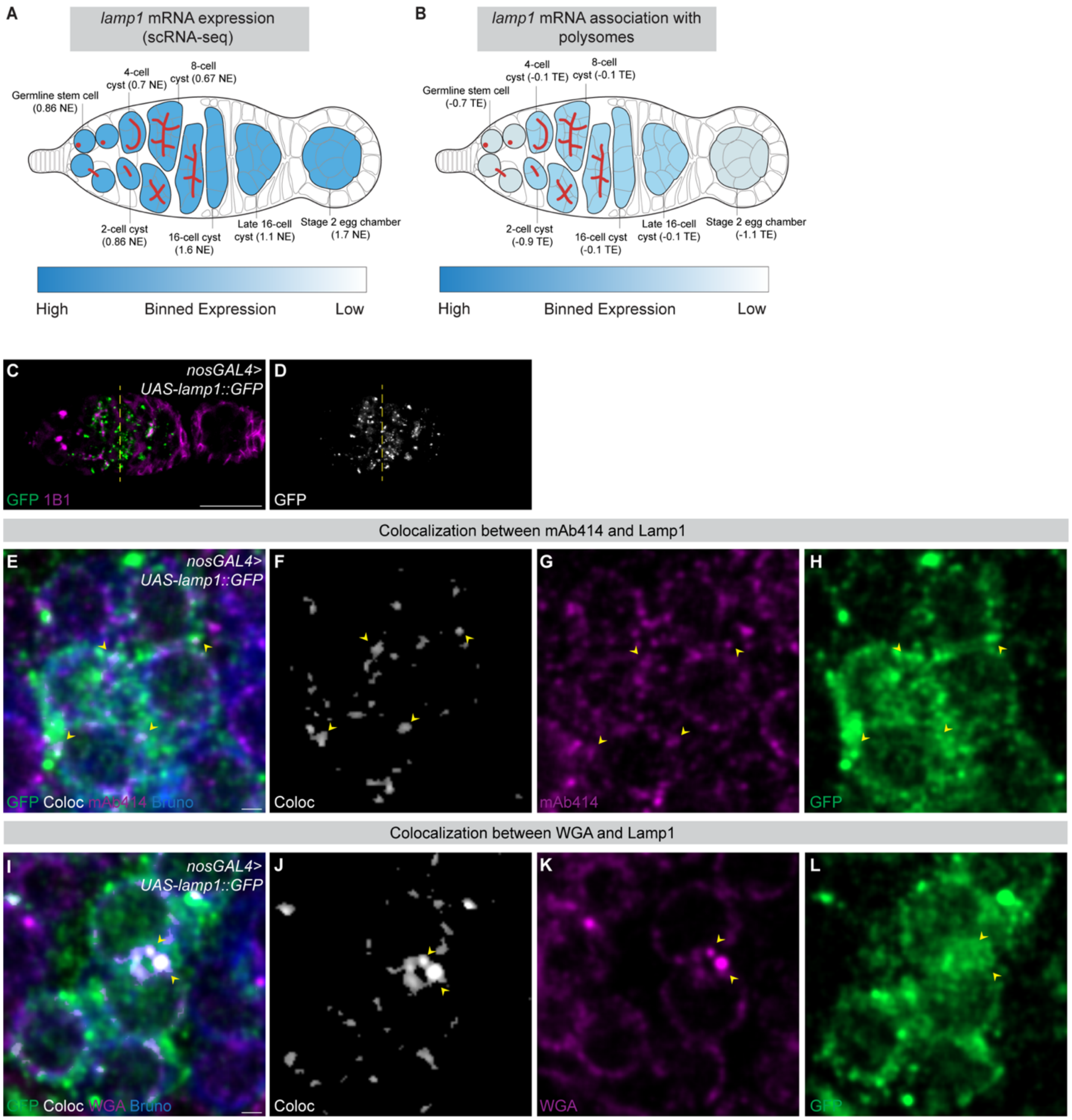

Venkat_fig.4S2

Fig. 4S2: Nucleoporins associate with Lamp1-positive vesicles during cyst differentiation. **(A–B)** Binned expression maps of *lamp1* mRNA abundance and *lamp1* mRNA association with polysomes across the germarium derived from scRNA-seq datasets. Dark blue indicates higher expression/association, and light blue indicates lower expression/association.
(C–D) Confocal images of germaria expressing *UAS–lamp1::GFP* driven by *nos*GAL4. GFP fluorescence indicates Lamp1-positive vesicles, which are enriched during cyst stages. Scale bar, 25 µm.

bioRxiv preprint doi: https://doi.org/10.64898/2026.02.13.705775; this version posted February 16, 2026. The copyright holder for this preprint (which was not certified by peer review) is the author/funder, who has granted bioRxiv a license to display the preprint in perpetuity. It is made available under aCC-BY 4.0 International license.

(E–H) Confocal images of an 8-cell cyst *from UAS–lamp1::GFP* germaria driven by *nos*GAL4, co-stained for Bruno to mark cyst stages and mAb414 to label NPCs. Yellow arrowheads indicate Lamp1-positive vesicles overlapping with NPC signal. Colocalization was assessed using Imaris 10.2 with intensity thresholds of 26 for mAb414 and 27 for GFP. Approximately 10.36% of Lamp1-positive vesicles overlapped with NPC signal. Scale bar, 5 μm.

(I–L) Confocal images of an 8-cell cyst from *UAS–lamp1::GFP* germaria driven by *nos*GAL4, co-stained for Bruno and WGA to label NPCs. Yellow arrowheads indicate Lamp1-positive vesicles overlapping with NPC signal. Colocalization was assessed using Imaris 10.2 with intensity thresholds of 26 for WGA and 27 for GFP. Approximately 17.34% of Lamp1-positive vesicles overlapped with NPC signal. Scale bar, 5 μm.

bioRxiv preprint doi: https://doi.org/10.64898/2026.02.13.705775; this version posted February 16, 2026. The copyright holder for this preprint (which was not certified by peer review) is the author/funder, who has granted bioRxiv a license to display the preprint in perpetuity. It is made available under aCC-BY 4.0 International license.

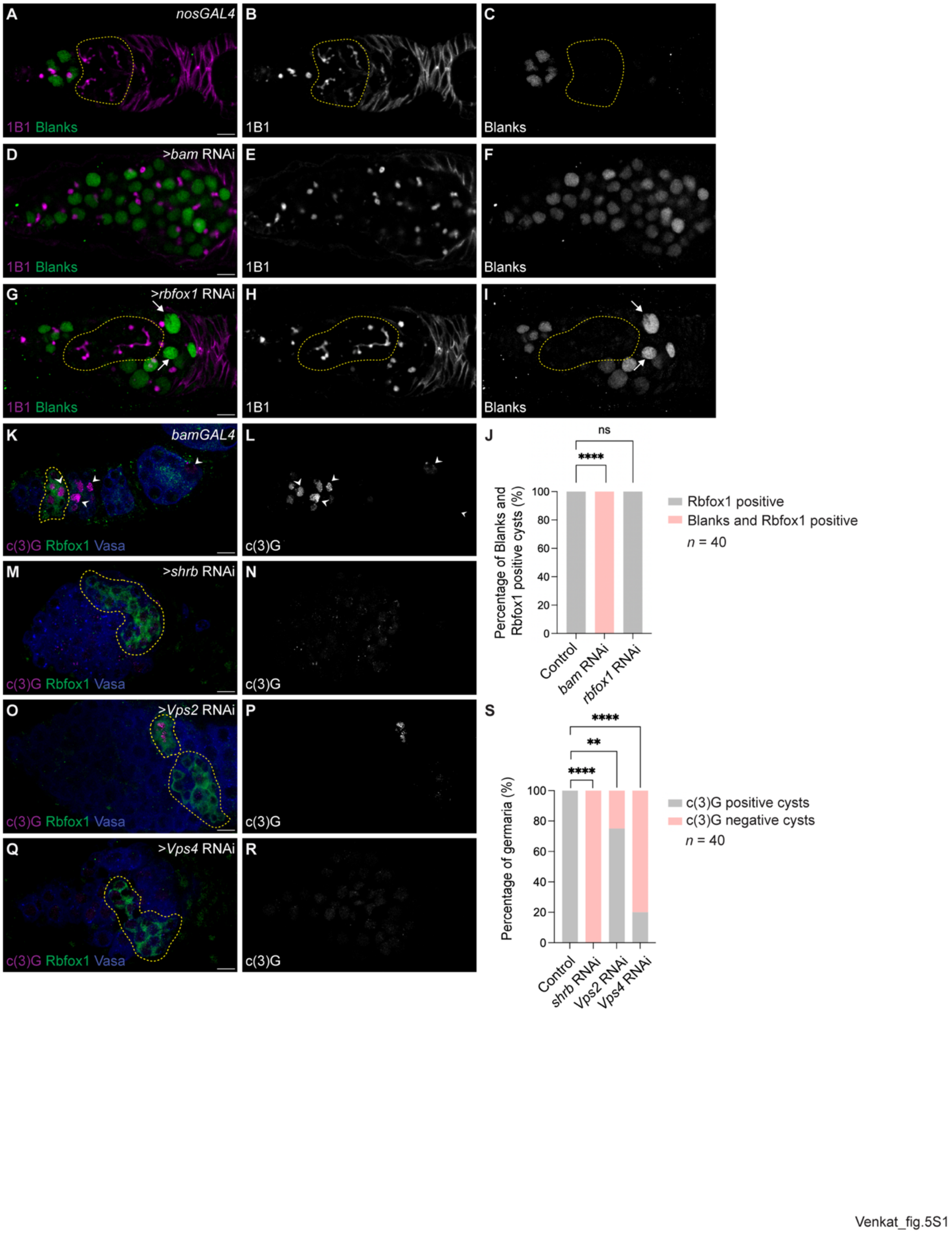

Figure 5S1: NPC degradation is required for initiation of meiotic recombination.
(A–I) Confocal images of control, *bam*-depleted and *rbfox1*-depleted stained for Blanks, a germ cell gene marker. *rbfox1* depletion results in accumulation of cysts, whereas *bam* depletion leads to accumulation of GSCs and cystoblasts. White arrows indicate Blanks-positive single germ cells. Yellow dashed outlines mark 8-cell cysts that do not express Blanks. Scale bars, 10 µm.
(J) Quantification of the percentage of germaria exhibiting extended Blanks expression in control, *bam*-, and *rbfox1*-depleted ovaries. Statistical analysis was performed using Fisher's exact test. *n* = 40 germaria per

(which was not certified by peer review) is the author/funder, who has granted bioRxiv a license to display the preprint in perpetuity. It is made available under aCC-BY 4.0 International license.

genotype; ****, $p < 0.0001$.
(K–R) Confocal images of control and germline-specific depletion of *shrb*, *Vps2*, or *Vps4* in germaria stained for crossover suppressor on 3 of Gowen [c(3)G], a marker of synaptonemal complex assembly and meiotic entry. c(3)G-positive cysts are observed in control germaria (white arrowheads) but are absent following depletion of *shrb*, *Vps2*, or *Vps4*. Yellow dashed outlines mark Rbfox1-positive 8-cell cysts. Scale bars, 10 μm.
(S) Quantification of the percentage of germaria containing c(3)G-positive cysts in control and *shrb*-, *Vps2*-, or *Vps4*-depleted ovaries. Statistical analysis was performed using Fisher's exact test. $n = 40$ germaria per genotype; ****, $p < 0.0001$; **, $p = 0.0010$.

bioRxiv preprint doi: https://doi.org/10.64898/2026.02.13.705775; this version posted February 16, 2026. The copyright holder for this preprint (which was not certified by peer review) is the author/funder, who has granted bioRxiv a license to display the preprint in perpetuity. It is made available under aCC-BY 4.0 International license.

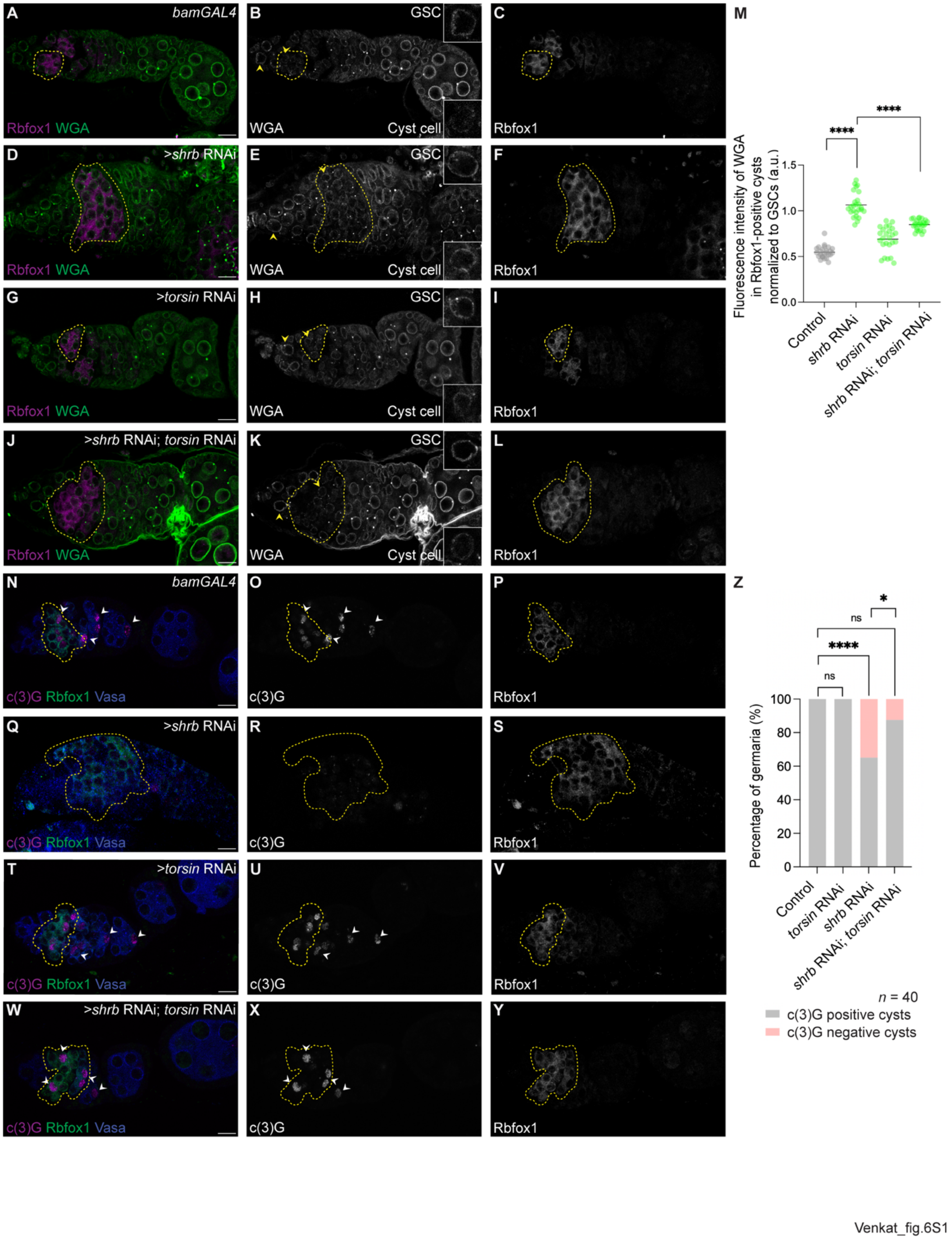

Fig. 6S1: Forced NPC reduction during cyst differentiation rescues meiotic recombination defects caused by loss of ESCRT-III.

(A–I) Confocal images of control, *shrb*-depleted, and *torsin*-depleted germaria stained with WGA to label NPCs and Rbfox1 to identify cyst stages. GSCs are indicated by yellow arrowheads.

bioRxiv preprint doi: https://doi.org/10.64898/2026.02.13.705775; this version posted February 16, 2026. The copyright holder for this preprint (which was not certified by peer review) is the author/funder, who has granted bioRxiv a license to display the preprint in perpetuity. It is made available under aCC-BY 4.0 International license.

(J–L) Confocal images of *shrb* and *torsin* double-depleted germaria showing reduced NPC signal in cysts following forced NPC reduction during cyst differentiation. GSCs are indicated by yellow arrowheads. **(M)** Quantification of WGA fluorescence intensity in Rbfox1-positive cysts, normalized to GSCs within the same germarium and expressed in arbitrary units (a.u.). Statistical analysis was performed using a two-tailed unpaired Welch's *t*-test. $n = 5$ germaria per genotype; ****, $p < 0.0001$. **(N–V)** Confocal images of control, *shrb*-depleted, and *torsin*-depleted germaria stained for c(3)G to assess initiation of meiotic recombination. White arrowheads indicate c(3)G-positive cells. **(W–Y)** Confocal images of germaria with combined depletion of *shrb* and *torsin* restores initiation of meiotic recombination in the *shrb*-depleted background. **(Z)** Quantification of the percentage of germaria containing c(3)G-positive cysts in control, *shrb*-, *torsin*-, and *shrb*/*torsin* double-depleted ovaries. Statistical analysis was performed using Fisher's exact test. $n = 40$ germaria per genotype; ns, $p > 0.9999$; *, $p = 0.0339$; ****, $p < 0.0001$.

Yellow dashed outlines mark Rbfox1-positive 8-cell cysts. Scale bars, 10 µm.

bioRxiv preprint doi: https://doi.org/10.64898/2026.02.13.705775; this version posted February 16, 2026. The copyright holder for this preprint (which was not certified by peer review) is the author/funder, who has granted bioRxiv a license to display the preprint in perpetuity. It is made available under aCC-BY 4.0 International license.

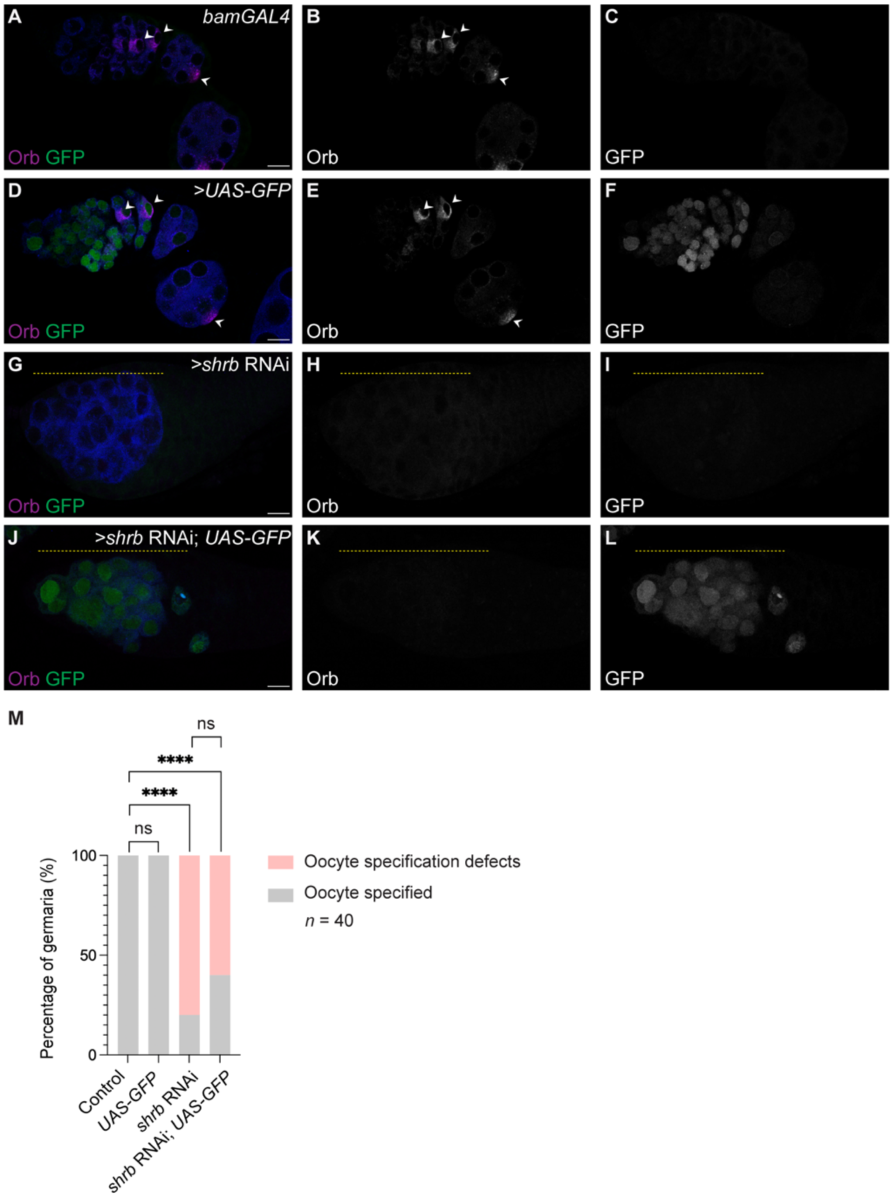

Venkat_fig.6S2

Figure 6S2: Addition of an extra UAS transgene does not rescue oocyte specification defects caused by loss of *shrb*.

(A–I) Confocal images of control germaria, germaria expressing *UAS–GFP* under *bamGAL4* control, and *shrb*-depleted germaria, stained for Orb and GFP to assess oocyte specification. In *shrb*-depleted germaria, Orb fails to localize to a single cell within the cyst (yellow dashed outlines), indicating defective oocyte specification. In contrast, proper Orb localization to a single cell is observed in control germaria and in germaria expressing UAS–GFP alone (white arrowheads).

(J–L) Confocal images of germaria expressing both *UAS–GFP* and *shrb* RNAi under *bamGAL4* control. Orb fails to localize to a single cell, indicating that the presence of an additional UAS transgene does not rescue the

bioRxiv preprint doi: https://doi.org/10.64898/2026.02.13.705775; this version posted February 16, 2026. The copyright holder for this preprint (which was not certified by peer review) is the author/funder, who has granted bioRxiv a license to display the preprint in perpetuity. It is made available under aCC-BY 4.0 International license.

oocyte specification defect caused by *shrb* depletion.

(M) Quantification of the percentage of germaria exhibiting a specified oocyte in control, *UAS–GFP–*expressing, *shrb*-depleted, and *UAS–GFP;shrb RNAi* germaria. Statistical analysis was performed using Fisher's exact test. n = 40 germaria per genotype; ns, p > 0.9999; ****, p < 0.0001.

Scale bars, 10 µm.

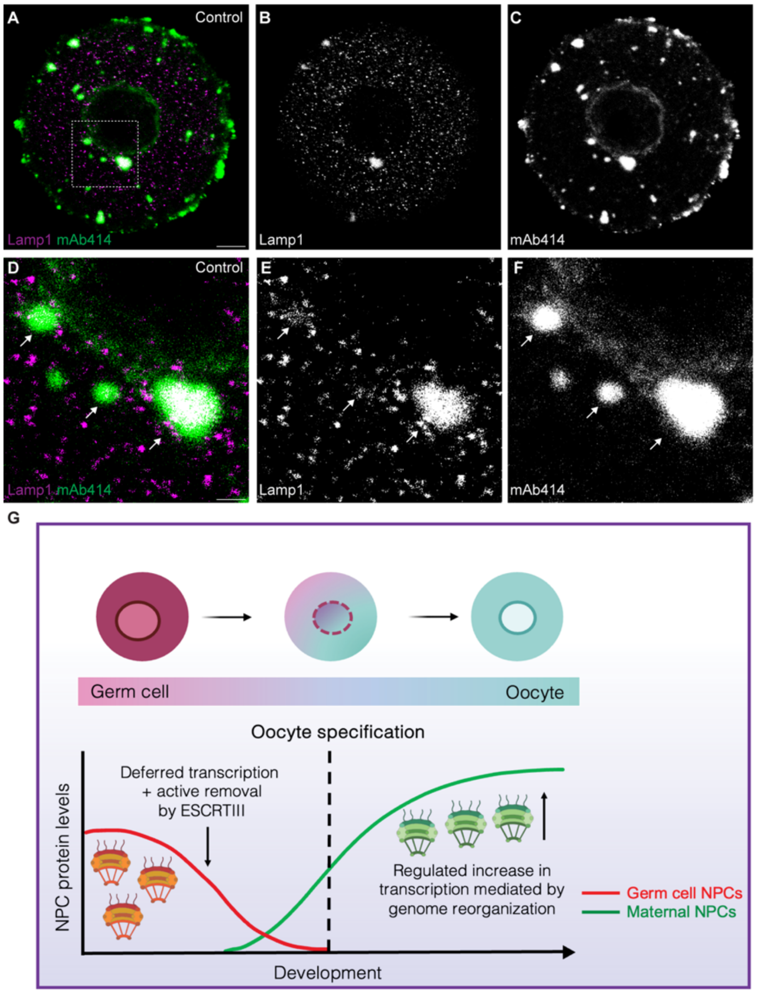

Venkat_fig.6S3

Figure 6S3: Nuclear pore complexes associate with lysosomes in prophase I–arrested mouse oocytes.
(A–C) Confocal images of prophase I–arrested murine oocytes stained with mAb414 to label NPCs and Lamp1 to mark lysosomes.

(D–F) Higher-magnification views highlighting regions of overlap between NPC and lysosome signals adjacent to the nuclear envelope (arrows). 100% overlap of puncta ~4µm (n = 5 oocytes). Scale bars, 12.5 µm.

(G) Model illustrating developmentally programmed replacement of germ cell NPCs with maternal NPCs during oocyte specification. Germ cell NPCs are reduced through a combination of passive dilution driven by deferred

bioRxiv preprint doi: https://doi.org/10.64898/2026.02.13.705775; this version posted February 16, 2026. The copyright holder for this preprint (which was not certified by peer review) is the author/funder, who has granted bioRxiv a license to display the preprint in perpetuity. It is made available under aCC-BY 4.0 International license.

nucleoporin transcription and active removal from the nuclear envelope mediated by the ESCRT-III/Vps4 pathway. Subsequent upregulation of nucleoporin transcription supports assembly of maternal NPCs, which promote large-scale genome reorganization and establishment of the maternal chromatin state.

Supplementary Table 1. RNA-seq and polysome-seq analysis of nucleoporin (Nup) transcripts. RNA-seq and polysome profiling–coupled RNA sequencing (polysome-seq) data for 29 nucleoporin genes analyzed in ovaries.
